# Cholinergic Interneurons in the Accumbal Shell Region Regulate Binge Alcohol Self-Administration in Mice: An In Vivo Calcium Imaging Study

**DOI:** 10.3390/brainsci14050484

**Published:** 2024-05-10

**Authors:** Rishi Sharma, Abigail Chischolm, Meet Parikh, Deepak Kempuraj, Mahesh Thakkar

**Affiliations:** Harry S. Truman Memorial Veterans Hospital, Department of Neurology, University of Missouri, Columbia, MO 65201, USA; sharmar@umsystem.edu (R.S.); ajc7ng@missouri.edu (A.C.); meetparikh@missouri.edu (M.P.); dkx2c@missouri.edu (D.K.)

**Keywords:** cholinergic interneurons, alcohol, nucleus accumbens, binge drinking, mice

## Abstract

Recently, we and others have shown that manipulating the activity of cholinergic interneurons (CIN) present in the NAc can modulate binge alcohol consumption. The present study is designed to examine the relationship between binge alcohol consumption and the activity of the CIN in real time by using an in vivo microendoscopic technique. We hypothesized that mice exposed to Drinking in the Dark (DID)—a recognized mouse model for binge drinking—would exhibit increased activity in the accumbal shell region (NAcSh). To test this hypothesis, male mice expressing Cre-recombinase in the cholinergic neurons were exposed to binge alcohol consumption (alcohol group), employing the DID method, and utilized in vivo calcium imaging to observe CIN activity in real time during alcohol consumption. The control (sucrose) group was exposed to 10% (*w*/*v*) sucrose. As compared to sucrose, mice in the alcohol group displayed a significant increase in the frequency and amplitude of discharge activity, which was measured using calcium transients in the CIN present in the NAcSh. In summary, our findings suggest that the activity of CIN in the NAcSh plays a crucial role in alcohol self-administration. These results emphasize the potential significance of targeting CIN activity as a therapeutic approach for addressing AUD.

## 1. Introduction

Cholinergic interneurons (CIN) are a major source of acetylcholine (ACh) in the nucleus accumbens (NAc) and have recently garnered attention as being a critical cellular component mediating the behavioral response to rewarding stimuli. Under normal conditions, these interneurons display a tonic activity and are hence often referred to as “tonically active neurons” (reviewed in [1]); however, in the presence of rewarding stimuli, such as cocaine, these neurons ramp up their firing patterns to facilitate reward collection [2,3]. In addition, CIN activity facilitates dopamine release through the activation of dopamine varicosities—specialized sites along dopaminergic axons in which neurotransmitter release occurs—and enhances the motivation to obtain a reward [4,5,6,7]. In the NAc, as compared to GABAergic medium spiny neurons (MSNs; >95%), the number of CIN is small (1–3%); however, with their extensive dendritic and axonal arborizations, the CIN acts as a major regulator of GABAergic synaptic transmission in the NAc [8,9].

Accumulating evidence suggests that alcohol may affect the activity of the CIN, whereas changes in the CIN activity may affect alcohol consumption. For example, the activation of CIN increases binge alcohol consumption in mice, while the genetic ablation of these cells leads to a reduction in consumption [10,11]. In addition, acute alcohol administration has been shown to increase the expression of activity marker c-Fos in the CIN, whereas chronic exposure to alcohol causes neuroplastic changes in the CIN, which may be responsible for facilitating alcohol drinking and relapse [12,13]. However, direct evidence establishing a link between alcohol self-administration and changes in the activity of CIN in the NAc is lacking. In our study, we sought to fill this gap by hypothesizing that mice engaged in binge drinking would demonstrate escalated discharge activity of CIN within the NAc. To test this hypothesis, our research employed in vivo calcium imaging techniques, enabling real-time monitoring of CIN activity while animals engaged in binge alcohol consumption via a well-established mouse model of binge drinking, the Drinking in the Dark (DID) method. The DID method has a high face validity in terms of mimicking human binge drinking. In this paradigm, C57BL/6J mice consume large amounts of alcohol voluntarily, without any prior training, resulting in a pharmacologically relevant blood alcohol concentration (BAC) within a short period of time. Moreover, the DID method is an ideal tool for studying the neurobiology underlying binge drinking [14,15,16,17].

## 2. Materials and Methods

All experiments were in accordance with the Association for Assessment and Accreditation of Laboratory Animal Care policies and the Guide for the Care and Use of Laboratory Animals. Animals: This study employed male transgenic mice, specifically B6J.129S6-Chat < tm2(cre)Lowl > /MwarJ, commonly known as ChAT-cre mice. These mice express Cre-recombinase under the control of the choline acetyltransferase (ChAT) promoter. The breeding pair of ChAT-cre mice, which were homozygous, was acquired from Jackson Laboratories, located in Bar Harbor, MA, USA (RRID: IMSR_JAX:028861). Following their procurement, the breeding of ChAT-cre mice was continued locally within the vivarium situated at the Harry S. Truman Memorial Veterans Hospital. The colony of mice was housed and maintained in adherence to standard vivarium conditions. The ambient room temperature was regulated and maintained at 25 ± 2 °C. The mice were exposed to a 12 h light/dark cycle, with lights being turned on at 7:00 A.M. each day. Moreover, the mice were granted unrestricted access to both food and water throughout this study, following an ad libitum feeding regimen.

### 2.1. Chemicals and Drugs

An alcohol solution (20% *v*/*v*) was prepared by mixing 200-proof absolute alcohol (Cat#B28184; Fisher Scientific, Pittsburgh, PA, USA) with water. Additionally, a sucrose solution (10% *w*/*v*) was prepared by dissolving sucrose (D-sucrose; Fisher Scientific) in water. For real-time micro-endoscopic in vivo imaging of cholinergic interneurons (CIN), adeno-associated virus (AAV; serotype 1) was employed, utilizing the FLEX switch system coupled with the calcium indicator GCaMP6s under the regulation of the human synapsin 1 promoter (GCaMP; AAV1.Syn.Flex.GCaMP6s.WPRE.SV40; 2 × 1013 GC/mL, diluted to 50%; Addgene, Cambridge, MA, USA). GCaMP, which stands for genetically encoded calcium indicator, is composed of several components, including the calcium-binding protein calmodulin (CaM), a peptide sequence derived from myosin light-chain kinase known as M13, and green fluorescent protein (GFP). When there are fluctuations in intracellular free calcium caused by neuronal activity, it triggers changes in the structure of the CaM–M13 complex, resulting in increased fluorescence emitted by GFP. This mechanism allows for the observation of neuronal activity in vivo using microscopy. All viral preparations were promptly aliquoted upon receipt and stored at −80 °C in a deep freezer. Moreover, all solutions were freshly prepared immediately before usage.

### 2.2. Surgeries for Intra-NAcSh Delivery of Calcium Indicator (GCaMP)

One week before the surgery, mice (5–6 weeks old), were transferred from the vivarium to the experimental room (similar conditions, except reverse 12 h light/dark cycle with lights off at 10:00 A.M.) and allowed to habituate to the new environment with ad libitum access to food and water. The animals underwent two invasive stereotaxic surgeries in separate sessions, allowing a 1- to 2-week interval between procedures.

The protocol is as follows: Under aseptic conditions and isoflurane anesthesia (1–2%), a burr hole was drilled in the skull. Initially, a 300 to 500 μm tissue section was aspirated using a 30-gauge blunt needle attached to a vacuum pump, followed by continuous saline application to prevent tissue dryness, and any bleeding was managed with sterile saline-soaked gel foam. Following tissue preparation, mice received microinjections of GCaM or cGCaM in the NAcSh (500 nL @ 100 nL/min). The co-ordinates used for microinjections were as follows: anterior (+1.3 mm), lateral (±1.0 mm), and ventral (−4.8 mm), relative to bregma and the skull surface [18]. Subsequently, a gradient refractive index lens (GRIN) lens (600 μm diameter, 7.3 mm long, Inscopix, Palo Alto, CA, USA) was slowly descended to approximately 200 μm above the targeted area, securing optical access. The exposed lens was fixed, anchored with skull screws, and coated with a silicon elastomer for protection.

A week later, a baseplate docking station (Inscopix, Palo Alto, CA, USA) was secured above the GRIN lens to support the miniaturized microscope on the animals’ heads as follows: The mice were anesthetized with isoflurane, and the silicone mold surrounding the lens was delicately removed using forceps (Fine Science Tools, Foster City, CA, USA). Any debris present on the exposed lens was cleared using compressed air, and a lens paper (Thorlabs Inc., Newton, NJ, USA) soaked in 70% alcohol was employed to clean the lens’s surface. Subsequently, the miniature microscope (2 g, maximum field of view 600 × 800 μm, single-channel epifluorescence: 475-nm blue LED; Inscopix), along with the attached baseplate, was carefully placed over the implanted lens using a microscope gripper (Inscopix). This involved aligning the microendoscope parallel to the lens by manually adjusting the scope’s tilt along the x and y axes and focusing the field of view using specific landmarks, such as clearly visible blood vessels or cells. Once the field of view was accurately focused and the scope was aligned parallel to the lens, the magnetic baseplate was fixed securely around the microendoscope with metabond, which was followed by covering the entire head cap with black nail polish to prevent light leakage. Subsequently, a protective cover was secured on the baseplate. Once ambulatory, the animals were left undisturbed for one week.

### 2.3. Habituation

One week after the implantation of the baseplate, the animals underwent a habituation procedure that spanned three days. This period aimed to familiarize the animals with the experimental environment and conditions. The procedure is as follows: Starting 2.5 h after the onset of darkness, the protective cover was removed from the baseplate. A “dummy” scope (Inscopix) resembling the actual microscope in both shape and weight was affixed onto it by gently restraining the animal without anesthesia. The animals were left undisturbed for 4 h, replicating the conditions experienced during the actual imaging sessions, while they were provided with water instead of alcohol or sucrose. After 4 h, the dummy microscope was removed, and the baseplate was covered. The same procedure was repeated for 2 more days.

### 2.4. Baseline Recording

Following 14 days of viral vector injection and a three-day habituation phase, a baseline imaging session was conducted within a single day. This session served as a reference point to establish the initial neuronal activity under normal conditions. Once habituated, a baseline imaging session was conducted in which an integrated microscope was utilized, instead of a “dummy” microscope, to capture images of calcium fluorescence in NAcSh CIN neurons through an implanted GRIN lens (described below). Subsequently, the animals were exposed to a binge alcohol consumption protocol.

### 2.5. Binge Drinking

To facilitate binge drinking, a well-established four-day Drinking in the Dark (DID) procedure was used as described previously [14]. In brief, starting 2.5 h after dark onset, water bottles were removed from each mouse cage. A total of 30 min later, 20% (*v*/*v*) alcohol (alcohol group; N = 4/group) or 10% (*w*/*v*) sucrose (sucrose group; N = 4/group) was dispensed in a pre-weighed bottle (identical to the water bottle) to each mouse in place of the water bottle. Mice were allowed to consume alcohol (or sucrose) for 2 h. On completion, the alcohol/sucrose bottles were removed and weighed, followed by weighing the animals to calculate the amount of alcohol/sucrose consumed (g/kg of the body weight). Subsequently, mice were re-provided with the original water bottle and left undisturbed. The same procedure was repeated on days 2 and 3. On day 4, the animals were allowed to consume alcohol or sucrose for 4 h.

### 2.6. Calcium Imaging Procedure

On day 4, starting from 2.5 h after the dark onset, the integrated microscope was attached to the baseplate. Grayscale HDF5 images were acquired at a rate of 10 frames per second, with an average exposure time of 100 milliseconds, using nVoke Acquisition Software (version 2.1, Inscopix). The analog gain on the image sensor was set between 1.0 and 3.0 while maintaining the EX-LED within the range of 0.5–1.2 mW/mm^2^ (measured from the bottom of the objective). Subsequently, calcium fluorescence was recorded via nVoke data acquisition software (Inscopix) at a rate of 10 frames per second. The recording began 3 h after the dark onset and was continued for a subsequent 4 h (18 min every hour). These recording parameters were determined through preliminary studies to minimize photobleaching while ensuring the adequate detection of fluorescent activity.

### 2.7. Processing and Analysis of Calcium Imaging Videos

The Inscopix Data Processing Software (Mosaic v. 1.9; Inscopix) was employed to process and interpret the acquired calcium imaging data from the Inscopix miniature microscopes (miniscope; nVoke; Inscopix). Before processing the imaging data, the region of interest (ROIs) was identified, selected, and cropped. To expedite processing, spatial down-sampling (8×) was performed without temporal down-sampling. This was followed by performing motion correction, which typically employs algorithms designed to align or correct for motion-related artifacts in the recorded calcium imaging data. Next, the ΔF/F0, a cell identification algorithm or operation to measure cell activity in a mini-scope movie, was applied to create a baseline-adjusted movie by normalizing the overall frame’s average fluorescence against the mean z-projection image (F0). ΔF was calculated by measuring the difference between the actual fluorescence during neuronal activity (F) and the baseline fluorescence (F0), indicating changes in fluorescence compared to the baseline level.

After generating the baseline-adjusted movie, manual contouring using a polygon tool was performed on the microscope movie to isolate and identify distinctive calcium transients. Manual identification was preferred over methodologies such as principal component analysis (PCA), independent component analysis (ICA), and constrained non-negative matrix factorization (CNMFe), as previously described [19,20,21], due to the limited number of CIN present in the NAcSh (<25 per ROI). Once selected, the algorithm generated a “Field Of View” displaying the location of each individual cell. With user-defined contours over the baseline-adjusted movie and changes in each cell fluorescence compared to the baseline level, an “Activity Map” was created along with a set of “Cell Traces”, with an *x*-axis showing the time in seconds and a *y*-axis showing the amplitude of the fluorescence intensity. On the activity map and traces, each cell is represented by a unique color outline or trace. Next, we used an event detection algorithm that evaluates a set of constraints, including negative transients, event threshold, and rate of decay, to automatically identify bursts of cell activity and extract the calcium-spiking events characterized by large-amplitude peaks (>two standard deviation) with rapid rises and gradual decay, with durations of at least 0.5 s, and separated from the adjacent calcium transient by >300 ms. The overarching goal was to identify cells with high signal-to-noise ratios and effectively isolate critical events in each unit (CIN) throughout the imaging duration. In each cell trace, these calcium transients detected using event detection algorithm can be visualized using the overlaid “dots”.

### 2.8. Data Analysis and Visualization

The calcium imaging data, encompassing both the maximum value of cell fluorescence linked to the burst firing of CIN and the frequency of cellular firing, was systematically observed and recorded over the entire recording duration. This recording duration spanned 18 min, capturing the initial phase of the first hour of both alcohol and sucrose consumption. The data obtained from these observations were diligently saved and stored on the local desktop computer for further analysis and evaluation. Moreover, this procedure was replicated for the subsequent 3 h period covering the remaining fluid consumption, ensuring consistency and continuity in the collection of imaging data.

Once the calcium imaging data during all 4 h of alcohol/sucrose consumption was recorded and saved, it was subsequently exported as .csv (Excel Version 2402) and .MP4 (video) files for offline analysis. Because mice consume the majority of alcohol during the first half of the 4 h alcohol access, we analyzed the data for only the first hour of alcohol/sucrose consumption, forming only one specific time point for evaluation. Z-scores were computed for each cell trace to identify calcium transients exhibiting a bell-shaped curve with distinct peak values. A “Raster plot” was created to visualize the occurrence and timing of calcium transients during alcohol or sucrose exposure. In the raster plot, each line signifies an individual calcium transient, with the *x*-axis representing time and the *y*-axis indicating different transients or cells [20]. A box plot was used to visualize the variability and distribution of calcium transient peaks and numbers observed during the entire 18 min session for both alcohol-exposed and sucrose-exposed animal groups.

### 2.9. Blood Alcohol Concentration

Blood alcohol concentration (BAC) was measured immediately at the end of alcohol consumption, as described previously [22]. Briefly, the mice were removed from their cages, and a small amount (25 µL) of blood was collected from the tail vein and centrifuged to separate plasma. The BAC was measured from the plasma using an alcohol measurement kit (Ethanol L3K^®^ Assay) as per the manufacturer’s instructions (Sekisui, Burlington, MA, USA).

### 2.10. Immunofluorescence

After the experimental procedures were concluded, mice were deeply anesthetized using CO_2_ and then perfused transcardially with ice-cold normal saline followed by 10% buffered formalin (fixative; Fisher Scientific). The entire head was severed and placed in 10% buffered formalin for two subsequent days. The brain was then extracted, further fixed overnight in the same fixative, and later treated with a solution of 20% sucrose (*w*/*v*) in 0.1 M phosphate-buffered saline with a pH of 7.4 until it reached equilibrium. Subsequently, the brain was sectioned into slices of 30 µm thickness, creating three series of sections using a freezing microtome [23,24]. One series of brain sections containing NAc was used to perform immunofluorescence using choline acetyltransferase (ChAT; SC-52; Santacruz Biotech, Dallas, TX, USA) antibody as the primary and Cy3-Donkey Anti-Rabbit IgG (H + L) (RED; 711-165-152; Jackson ImmunoResearch, West Grove, PA, USA) as the secondary antibody.

### 2.11. Microscopy and Quantification

For each animal, 4 sections containing CIN (RED) were used for bilateral counting the number of red fluorescent-labeled CIN and green fluorescent-labeled GCaM (GFP tag) expressed cells in the NAcSh area. Under 10× magnification and with the same focus, 2 photomicrographs were taken with the Thunder Imagers camera-based fluorescence microscope (Leica, Wetzlar, Germany) followed by computational clearing, a new opto-digital method to eliminate the out-of-focus blur [25], the first visualizing CIN neurons (red fluorescence) and the second visualizing GCaMP labeling (red fluorescence). Two photomicrographs were then merged and counted with Adobe Photoshop CS5 software (version 12). Double-labeled neurons (the presence of red and green or yellowish-green fluorescence) were considered CIN with GCAMP. Only red-fluorescent single-labeled neurons were considered CIN neurons without GCaMP. Only double-labeled CIN and GCaMP + ve neurons and single-labeled CIN neurons were counted. The counts were summed, and the percentage of CIN with GCAMP was calculated for each mouse and used for further analysis.

### 2.12. Statistical Design and Analysis

In this study, a total of 8 ChAT-cre mice were utilized and randomly allocated into two groups, namely, alcohol and sucrose, using an online GraphPad randomization calculator (weblink: https://www.graphpad.com/quickcalcs/randomselect1/ accessed on 3 May 2024) just before the experiment commenced. A total of 69 cells (15–18 cells/mouse) were recorded. Before conducting this study, a power analysis was conducted using G*Power (version 3.1), aiming for a sample size of N = 30 cells per group, with a significance level of α = 0.05 and a desired power of ≥0.9 [26] to determine effect size and sample size requirements.

All statistical analyses were performed using R statistical software (version 3.6.3, R Core Team, Vienna, Austria) [27]. Grubb’s test was applied to identify any potential outliers within the data. Furthermore, the Shapiro–Wilk normality test was conducted to assess the normal distribution of the collected data.

To determine the significant differences between the alcohol and sucrose groups regarding the peak fluorescence value associated with burst firing and the frequency of calcium transients, a linear mixed-effects model with nested analysis was utilized. In instances in which the data exhibited normal distribution, the Student *t*-test was applied. For instance, the data pertaining to the co-immunofluorescence of GCaMP6s and CIN underwent analysis using the Student *t*-test due to its normal distribution. The significance level (α) for all analyses was set at 0.05.

## 3. Results

### Localization of Lens Implantation Site and GCAMP6s Expression in CIN Neurons

**Figure 1:**
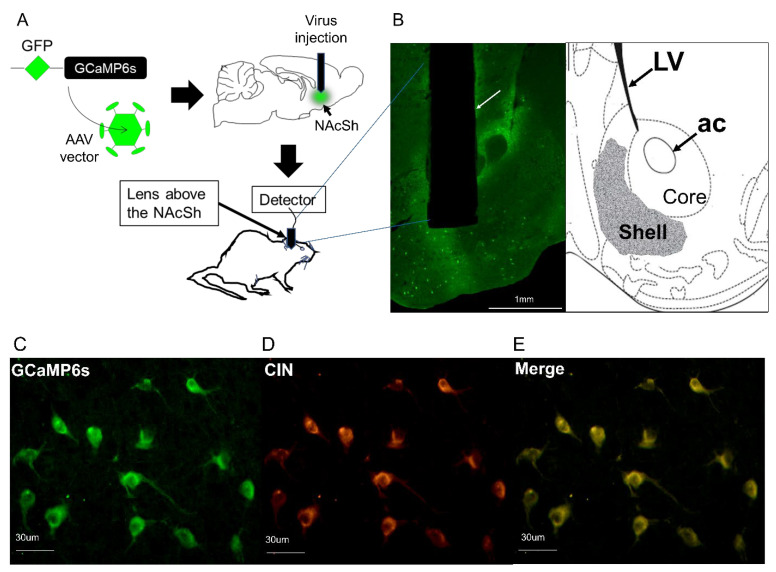
Localization of lens implantation site and the expression of GCAMP6s in CIN of NAcSh. (**A**) illustrates a schematic representation depicting the nucleus accumbens shell region (NAcSh) of ChAT-cre mice that were transfected with adeno-associated viruses (AAVs) containing cre-dependent GCaMP6s. This transfection resulted in the selective expression of GCaMP6s within the cholinergic interneurons (CIN) of NAcSh. (**B**) A representative photomicrograph displays a lesion (indicated by white arrow) resulting from the GRIN lens implantation within the nucleus accumbens shell (NAcSh), alongside neurons expressing green fluorescence attributed to the presence of GCaMP6s. Additionally, a coronal schematic (AP = 1.2), adapted from Figure 21 in [18], is included to provide further illustration of this precise localization. LV = lateral ventricles; ac = anterior commissure. (**C**–**E**) Representative photomicrograph showing green-fluorescent neurons (suggesting GCaMP6s expression; (**C**)) and red fluorescent neurons (suggesting ChAT expression in the CIN; (**D**)) and a merged image of (**C**,**D**) showing GCaMP6s expression in CIN (yellowish orange-fluorescent neurons; (**E**)).

The NAcSh of ChAT-cre mice was transfected with AAVs harboring cre-dependent GCaMP6s, causing this to express selectively in CIN (Figure 1A). Immunofluorescence was performed to identify the location of the GRIN lens and assess the co-expression of GCaMP6s and CIN. We found that the GRIN lens was consistently located in the NAcSh region, specifically between the coordinates AP  =  1.5 and 1.1, within the entire animal cohort (N = 8). Figure 1B displays a representative photomicrograph delineating the expression of green fluorescence in neurons, suggesting GCaMP6s expression, and the lesion resulting from the GRIN lens implantation within the NAcSh. Additionally, a coronal schematic (AP  =  1.2), adapted from Figure 21 in [18], is provided to further illustrate this precise localization.

In addition, co-immunofluorescence was performed, utilizing the ChAT antibody to label CIN in the NAcSh, and showed that there was no significant (t = 0.19; *p* = 0.85; Student *t*-test) difference in the percentage co-labeling between the groups. The mean ± SEM percentage co-labeling in the alcohol group was 85.3 ± 4.6 (N = 4), and the percentage co-labeling in the sucrose group was 86.6 ± 5.0 (N = 4). Overall, >85% of CIN neurons (cells expressing ChAT IF; red fluorescence) were co-labeled with GCAMP6s (green fluorescence) in the NAcSh. The representative photomicrograph is shown in Figure 1C–E.

(a) ***Alcohol consumption:*** In the alcohol group, mice displayed a consistent amount of alcohol consumption, with 3.4 ± 0.1 g/kg on day 1, 3.7 ± 0.2 g/kg on day 2, and 4.0 ± 0.1 g/kg on day 3, with no significant difference (F_2,9_ = 1.50; *p* = 0.27). On day 4, the mean ± SEM amount of alcohol consumption was 5.3 ± 0.1 g/kg, with a BAC of 102.6 ± 1.6 g/dL, suggesting binge alcohol consumption.
(b) ***Sucrose consumption:*** In the sucrose group, mice displayed a consistent amount of sucrose consumption, with 9.64 ± 0.031 g/kg on day 1, 9.95 ± 0.29 g/kg on day 2, and 10.11 ± 0.09 g/kg on day 3, with no significant difference (F_2,9_ = 0.94; *p* = 0.43). On day 4, the mean ± SEM amount of sucrose consumption was 13.88 ± 0.52 g/kg.
(c) Binge alcohol consumption increases the CIN activity in the NAcSh.

**Figure 2:**
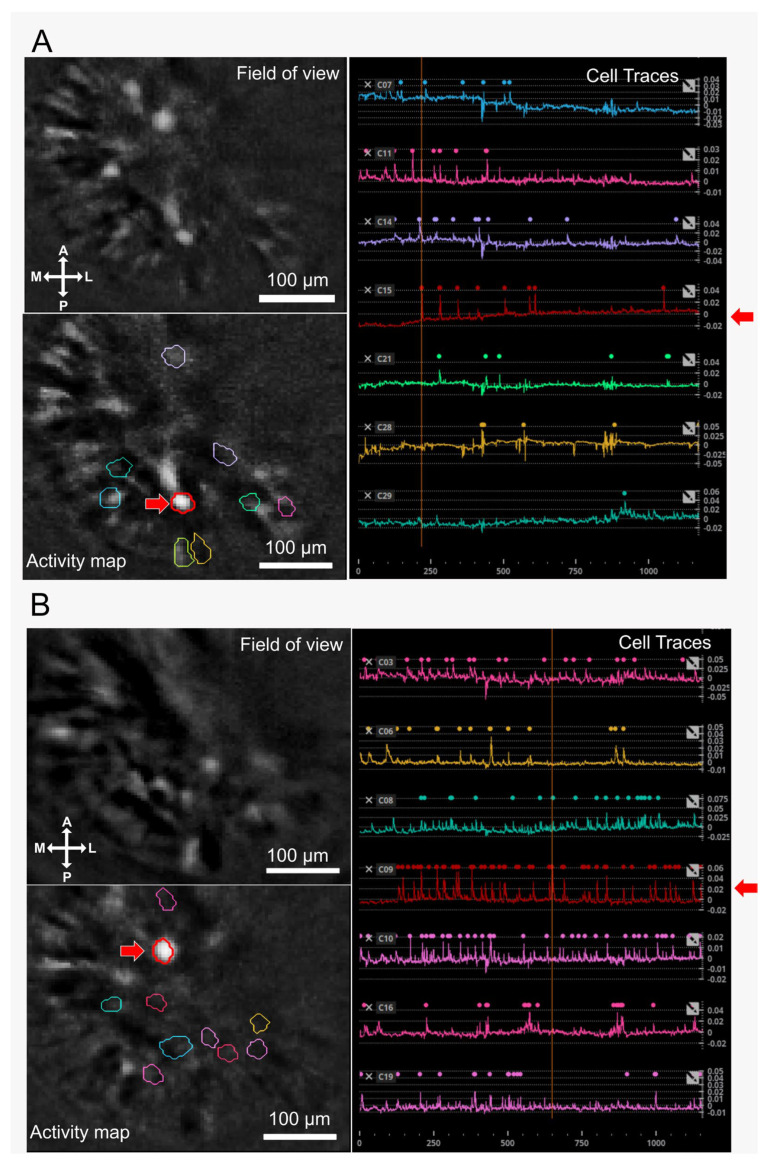
Discharge activity map and cell traces of cholinergic interneurons (CIN) in the shell region of nucleus accumbens (NAcSh) during binge alcohol consumption. In (**A**,**B**), a representative field of view displays the location of each individual cell extracted from recorded imaging videos (scale bar = 100 µm). An activity map within the same panel illustrates the location of selected cells highlighted by various colors. Additionally, cell traces corresponding to the selected cells in the activity map are presented using the same color code, with overlaid detected events showcasing individual calcium dynamics assessed across the session (18 min) for both the sucrose (**A**) and alcohol (**B**) groups. A red arrow in each panel highlights the discharge activity and trace of the same cholinergic interneuron (CIN) in the nucleus accumbens shell (NAcSh).

Video S1 (alcohol group) and Video S2 (sucrose group) show an example of an 18 min imaging session during alcohol or sucrose consumption on day 4. Figure 2 displays the representative field of view (location of each individual cell extracted from the recorded imaging videos), activity map, and cell traces with overlaid detected events (individual calcium dynamics assessed across the session) of sucrose (panel A) and alcohol (panel B) group.

**Figure 3:**
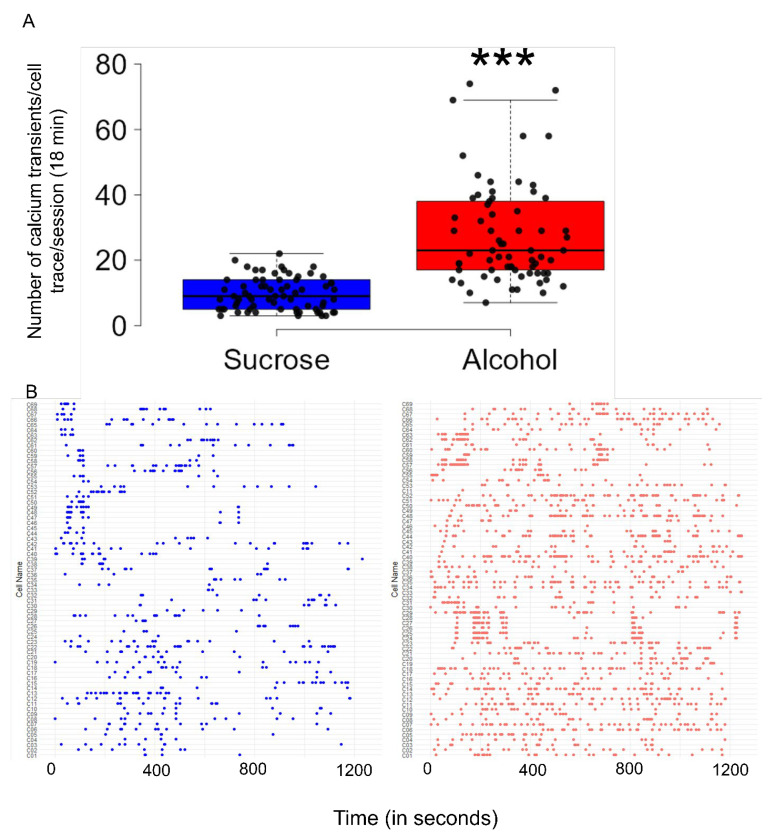
Amount of discharge activity of CIN in the NAcSh during binge alcohol consumption. Compared to sucrose consumption, the amount of discharge activity of CIN was significantly higher (*p* < 0.001) during binge alcohol consumption (**A**). The frequency of each recorded CIN in the NAcSh of mice exposed to sucrose and alcohol is displayed in the raster plot (**B**). *** *p* < 0.001 vs. Sucrose.

After implementing nested statistical analysis, which accounted for multiple cellular recordings from the same mice, a significant increase in the frequency of calcium transients (number of calcium peaks) was observed during alcohol consumption compared to sucrose (t = 7.77; df = 6.0; *p* < 0.001). The mean frequency ± SEM of calcium transients in the alcohol group was 28.3 ± 2.0, which was significantly higher than the 9.7 ± 0.6 observed in the sucrose group (Figure 3A). The distributions of individual calcium transients are displayed using a raster plot (Figure 3B).

There was no significant (t = 0.203; df = 6.0; *p* = 0.84) difference in the baseline number of calcium transients between alcohol (0.013 ± 0.0009) and sucrose (0.014 ± 0.0011) groups.

**Figure 4:**
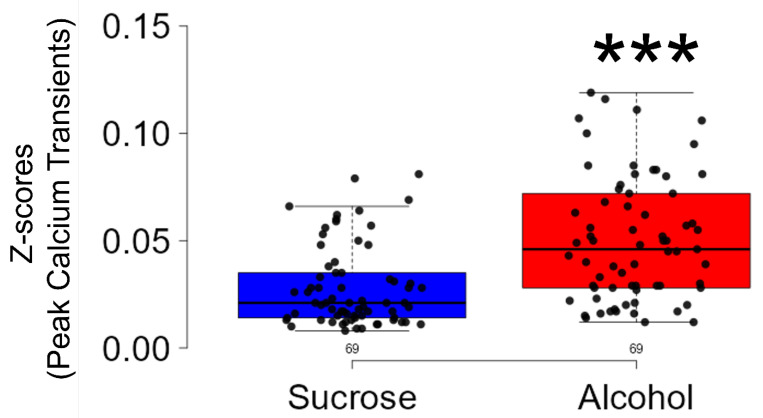
Peak discharge activity of CIN in the NAcSh during binge alcohol consumption. Compared to that of sucrose consumption, the amplitude of discharge activity of CIN was significantly higher (*p* < 0.001) during binge alcohol consumption. *** *p* < 0.001 vs. Sucrose.

In the linear mixed-effects model analysis for peak values of calcium transients, a significant difference was observed between the alcohol and sucrose groups (t = 2.774, df = 6.0; *p* < 0.01) (Figure 4). The mean ± SEM of the peak calcium transients or z-scores in the alcohol group was 0.05 ± 0.003, as compared to 0.034 ± 0.004 in the sucrose group. No significant (t = 0.290; df = 6.0; *p* = 0.78) difference was observed in the baseline peak values of the calcium transients between the alcohol (0.013 ± 0.0009) and sucrose (0.014 ± 0.0011) groups.

## 4. Discussion

Recently, we have shown that changes in the activity of CIN present in the NAcSh can modulate binge alcohol consumption. In the present study, we used an in vivo microendoscopic technique to examine the relationship between binge alcohol consumption and the activity of the CIN in real time. The significant findings of this study include (a) A significant increase in the firing rate of CIN in the NAcSh of C57BL/6J mice engaged in binge alcohol consumption compared to those in the sucrose group. This increase was assessed by observing calcium transient events in CIN during alcohol/sucrose consumption. (b) A significant rise in the amplitude of calcium transients, indicating the occurrence of calcium signals in phasic bursts, as evidenced by peak calcium transient activity during alcohol/sucrose consumption. Our findings suggest that CIN may play an important role in binge alcohol consumption.

Binge drinking is a prevalent and risky behavior characterized by consuming excessive amounts of alcohol within a relatively short time, resulting in a blood alcohol concentration (BAC) of 0.08 g/dL percent or higher [28]. Binge drinking is linked to 1 in 6 drug overdose deaths and contributed to the highest number of traffic-related fatalities in 2020 since 2008 [29]. During alcohol consumption, a surge in dopamine levels within the NAc induces a subjective sense of euphoria [30,31,32]. This intense pleasure reinforces repeated binge drinking, increasing the vulnerability to develop alcohol use disorders (AUD) [33,34]. Hence, apart from the immediate risks, binge drinking is strongly linked to the subsequent development of AUD among young individuals, leading to severe health, social, and occupational repercussions [35,36,37]. In the present study, we used the DID paradigm in C57BL/6J mice, a very well-known model with a high face validity in terms of mimicking human binge drinking. Moreover, we and others have used this method for studying the neurobiology underlying binge drinking [17,38,39,40,41].

Strong evidence suggests that NAc is the terminal output center of the mesolimbic dopamine system, which is comprised mainly of the rostral dopaminergic projection from the ventral tegmental area (VTA) to the NAc [33]. Although the activity of dopaminergic systems in the VTA is instrumental in alcohol-related dopamine release in the NAc [33], direct and indirect evidence strongly suggest that this increase in dopamine levels might be attributed to localized phenomena. Specifically, research highlights the role of CIN within the NAc in modulating dopamine release and contributing to reward and addiction mechanisms [11,12,13,42,43,44]. Indeed, while the burst firing in the CIN causes an increase, its inhibition reduces the frequency of dopamine transients in the NAc [4]. In addition, an increase in dopamine efflux in the NAc after the rewarding stimuli, including cocaine, is regulated by CIN [7]. While alcohol is a rewarding substance and CIN regulates dopamine release in the NAc, inducing pleasure in response to the rewarding stimuli, the relationship between binge alcohol consumption and in vivo real-time activity of CIN in the NAcSh has not been investigated.

We used in vivo real-time imaging of a genetically encoded calcium indicator (GCaMP6s) to examine CIN activity during voluntary binge alcohol consumption. In vivo calcium imaging allows for the targeted study of neurons in freely behaving animals, linking neural activity with behavior [20,45,46,47]. We utilized GCaMP6 as a fluorescent calcium indicator because it is known for producing strong fluorescence transients, even in response to individual action potentials. This characteristic enables high detection rates for single spikes. However, due to its slower decay time, GCaMP6 may not accurately capture high-frequency neuronal spikes and might not precisely reflect the actual firing rate of neurons compared to electrophysiology methods [48,49,50]. The NAc consists of two distinct subregions, the shell (NAcSh, a part of extended amygdala) and the core (NAcC, a part of dorsal striatum) [51,52]. While both have been implicated in the regulation of addictive behaviors, the NAcSh is crucial for binge or excessive alcohol consumption [31,53]. Studies indicate that CIN is present in a higher density in the NAcSh [31,39]. Hence, our study focused on studying the activity of CIN in the NAcSh during binge alcohol consumption.

Our investigation revealed a significant rise in both the frequency and amplitude of calcium transients recorded during periods of binge alcohol consumption compared to sucrose intake. Specifically, there was a substantial increase in the number of calcium transients (28.3 ± 2.0 in the alcohol group vs. 9.7 ± 0.6 in the sucrose group), indicating heightened neuronal activity during alcohol consumption. Additionally, the amplitude, reflecting the intensity of calcium transients exhibited a significant elevation during binge alcohol intake (0.05 ± 0.003), as compared to the intensity of calcium transients during sucrose consumption (0.034 ± 0.004). These findings underscore the robust CIN response (frequency and amplitude) associated with binge alcohol consumption and emphasize the significant differences in calcium transient dynamics in the CIN between alcohol and sucrose consumption. These results corroborate and further confirm previous findings that strongly support the role of CIN in modulating binge alcohol consumption [10,11,44].

We did not include a separate water control in our study; however, we have found a notable (200%) increase in the activity of CIN during sucrose administration (vs. water baseline). Additionally, even though the CIN activity was higher with sucrose (200%) as compared to water, the activity was specific to alcohol because the activity of CIN doubled in the presence of alcohol (vs. sucrose). In support of this CIN specificity, our recent study showed that the chemogenetic activation of CIN significantly reduced alcohol consumption without affecting sucrose consumption [11].

Research suggests that females may display a higher susceptibility to binge drinking [54]. While our study did not specifically investigate sex-specific differences in CIN activity, delving into such variations in binge drinking patterns and their influence on CIN function could offer valuable insights into the neurobiological mechanisms driving alcohol addiction across both male and female cohorts.

## 5. Conclusions

In summary, the results of this study suggest that CIN in the NAcSh display a substantial increase in both the frequency and amplitude of calcium transients during binge alcohol consumption. Moreover, this effect is specific to alcohol because the frequency and amplitude of these neurons were significantly lower during the consumption of sucrose, a natural reinforcer. Although we have not explored the neuroanatomical target downstream of CIN, it is possible that CIN interact with GABAergic MSNs in the NAcSh to affect drinking. These findings strongly implicate CIN as a potential therapeutic target for AUD, indicating its crucial role in modulating binge alcohol consumption.

## Data Availability

The data presented in this study are available on request from the corresponding author. The data are not publicly available due to privacy reasons.

## Supplementary Materials

Video S1: Alcohol Group.MP4 https://zenodo.org/records/10982353/files/S1-Alcohol%20Group.mp4?download=1; Video S2: Sucrose Group.MP4 https://zenodo.org/records/10982353/files/S2-Sucrose%20Group.mp4?download=1.
